# MicroRNA‐205‐5p targets E2F1 to promote autophagy and inhibit pulmonary fibrosis in silicosis through impairing SKP2‐mediated Beclin1 ubiquitination

**DOI:** 10.1111/jcmm.16825

**Published:** 2021-08-24

**Authors:** Qingzeng Qian, Qinghua Ma, Bin Wang, Qingqiang Qian, Changsong Zhao, Fumin Feng, Xiaona Dong

**Affiliations:** ^1^ School of Public Health North China University of Science and Technology Tangshan China; ^2^ Department of Preventive Health The Third People’s Hospital of Xiangcheng District in Suzhou Suzhou China; ^3^ Department of Pediatrics North China University of Science and Technology Affiliated Hospital Tangshan China; ^4^ Department of Neurology Tangshan Gongren Hospital Tangshan China; ^5^ Department of Emergency Tangshan Hospital of Traditional Chinese Medicine Tangshan China; ^6^ Department of Respiratory Medicine Tangshan People's Hospital Tangshan China

**Keywords:** Beclin1 ubiquitination, E2F1, macrophage autophagy, MicroRNA‐205‐5p, pulmonary fibrosis, silicosis, SKP2

## Abstract

Silicosis is an occupational disease characterized by extensive pulmonary fibrosis, and the underlying pathological process remains uncertain. Herein, we explored the molecular mechanism by which microRNA‐205‐5p (miR‐205‐5p) affects the autophagy of alveolar macrophages (AMs) and pulmonary fibrosis in mice with silicosis through the E2F transcription factor 1 (E2F1)/S‐phase kinase‐associated protein 2 (SKP2)/Beclin1 axis. Alveolar macrophages (MH‐S cells) were exposed to crystalline silica (CS) to develop an in vitro model, and mice were treated with CS to establish an in vivo model. Decreased Beclin1 and increased SKP2 and E2F1 were identified in mice with silicosis. We silenced or overexpressed miR‐205‐5p, E2F1, SKP2 and Beclin1 to investigate their potential roles in pulmonary fibrosis in vivo and autophagy in vitro. Recombinant adenovirus mRFP‐GFP‐LC3 was transduced into the MH‐S cells to assay autophagic flow. Knocking down Beclin1 promoted pulmonary fibrosis and suppressed the autophagy. Co‐immunoprecipitation and ubiquitination assays suggested that SKP2 induced K48‐linked ubiquitination of Beclin1. Furthermore, chromatin immunoprecipitation‐PCR revealed the site where E2F1 bound to the SKP2 promoter between 1638 bp and 1645 bp. As shown by dual‐luciferase reporter gene assay, the transfection with miR‐205‐5p mimic inhibited the luciferase activity of the wild‐type E2F1 3′untranslated region, suggesting that miR‐205‐5p targeted E2F1. Additionally, miR‐205‐5p overexpression increased autophagy and reduced the pulmonary fibrosis, while overexpression of E2F1 or SKP2 or inhibition of Beclin1 could annul this effect. The current study elucidated that miR‐205‐5p targeted E2F1, thereby inhibiting SKP2‐mediated Beclin1 ubiquitination to promote macrophage autophagy and inhibit pulmonary fibrosis in mice with silicosis.

## 1 INTRODUCTION

Silicosis is one of the most common occupational respiratory diseases that can manifest in respiratory failure or fatality in severe circumstances.^1^ The fundamental cause of the disease is the inhalation of crystalline silica (CS), which progresses to fibrosis in lung parenchyma.^2^ Fibrosis can evidently impair and affect the normal function of lung tissues for essential gas exchange and oxygen supply.^3^ Although effective prevention has been impactful, silicosis still persists as a global health concern, especially in China. In 2016, the statistics reported that a total of 27992 people suffered from pneumoconiosis, accounting for 88.06% of occupational diseases, with a detrimental increase of over 1000 cases than in 2015.^4^ Currently, in addition to the clinically adopted potential lung transplantation, extracellular vesicles and lung spheroid cells have been reported to serve as therapeutic modalities for lung regeneration, yet the clinical translation remains challenging currently, highlighting the urgent need for the development of other effective treatments.^5^, ^6^, ^7^ On account of these literature reports, the identification of vital molecules involved in silicosis is urgent and necessitated for improving the clinical outcome.

Autophagy is a highly conservative catabolic process, instrumental for tissue homeostasis, with vital functionality in regulating inflammation and apoptosis.^8^ Essentially, autophagy is a protective mechanism against different diseases, such as cancer, infectious diseases and lung injury.^9^, ^10^, ^11^ Additionally, existing evidence has ascertained the significance of autophagy in the development of silicosis.^12^ However, whether and how autophagy is involved in the progress of silicosis remain uncertain.

Beclin1 is the mammalian orthologue of yeast autophagy‐related gene‐6, regarded for involvement in the autophagy process.^13^ Its concentration increases under cellular stress, interacts with several cofactors and induces autophagy.^14^ Beclin1 was reported to affect the silicosis progression through regulation of autophagy of alveolar macrophages (AMs).^15^ Accordingly, the manifestation of reduced autophagic flux in alveolar epithelial cells was regarded to be crucial for CS‐induced silicosis, leading to apoptosis and pulmonary fibrosis.^16^

Increasing evidence has ascertained the deregulation of microRNAs (miRNAs) in various pathological processes such as pulmonary fibrosis and heart failure.^17^, ^18^ Changes in miRNA expression may be consequent for alteration of transcriptional activity of genes in immune‐mediated lung disease and are therefore closely associated with respiratory diseases such as silicosis.^19^ An existing study reported that miR‐1224‐5p affects CS‐induced pulmonary fibrosis by targeting Beclin1.^20^ After bioinformatics analysis, we identified that miR‐205‐5p might be an upstream miRNA regulating E2F transcription factor 1 (E2F1), which could potentially be the transcription factor of S‐phase kinase‐associated protein 2 (SKP2). As SKP2 has been reported to mediate the ubiquitination of Beclin1,^21^ we speculated the involvement of miR‐205‐5p in autophagy and pulmonary fibrosis through mediating the E2F1/SKP2/Beclin1 axis.

To testify this hypothesis, we performed in vitro and in vivo experiments in MH‐S cells and mice with silicosis. Our findings revealed that miR‐205‐5p impaired pulmonary fibrosis and E2F1/SKP2/Beclin1 axis was responsible for the role of miR‐205‐5p in such process. Therefore, miR‐205‐5p may be a promising target for the development of treatment protocols to inhibit silicosis progression.

## 2 METHODS

### 2.1 Ethical statement

The study involving human beings was conducted with approval of the Ethics Committee of Tangshan People's Hospital. The informed consents were obtained from all the patients and donors or their legal guardians prior to our investigation. The procedures were conducted in strict accordance with the *Declaration of Helsinki*. The use of the clinical cases was approved by the Committees of Animal Experimental Safety and the Animal Welfare Ethical Review Board of the college. Animal protocols were conducted with approval of the animal experimentation ethics committee of Tangshan People's Hospital.

### 2.2 Bioinformatics analysis

The pneumosilicosis fibrosis‐related gene expression microarray (GSE110711) data were obtained from the Gene Expression Omnibus (GEO) database (https://www.ncbi.nlm.nih.gov/gds), after which the ‘edgeR’ software package in R language was adopted for differential analysis. In microarray data set GSE110711, 3 normal untreated mouse samples and 3 silicosis model mouse samples were screened for the significant differential genes with |logFoldChange| >1, and *p* < 0.05 set as the threshold. The target genes of miR‐205 were predicted using a combination of the database starBase (http://starbase.sysu.edu.cn/index.php), TargetScan (http://www.targetscan.org/vert_71/) and MicroT_CDS (http://diana.imis.athena‐ innovation.gr/DianaTools/index.php?r=MicroT_CDS/index). A list of the mouse transcription factors was provided by AnimalTFDB (http://bioinfo.life.hust.edu.cn/AnimalTFDB/#!/). The target gene of transcription factor E2F1 was predicted using the database hTFtarget (http://bioinfo.life.hust.edu.cn/hTFtarget#!/).

### 2.3 Clinical samples

Clinical samples including the diseased tissues from silicosis patients and normal tissues from healthy organ donors (*n* = 10) were collected from the Tangshan People's Hospital. All diagnoses were confirmed by histopathological assessment with haematoxylin‐eosin (HE) and Masson staining.^22^

### 2.4 Immunohistochemistry (IHC) and histological analyses

For IHC, the clinical tissue paraffin sections were subjected to antigen retrieval, blocked using goat serum (C‐0005, Shang Hai Haoran Biological Technology Co., Ltd.,) and finally incubated with the corresponding primary antibodies, including rabbit anti‐human Beclin1 (ab210498, 1: 100, Abcam Inc.), E2F1 (ab179445, 1: 50, Abcam), or SKP2 (ab68455, 1: 500, Abcam) and light chain 3 (LC3) (ab48394, 1: 400, Abcam), respectively overnight at 4°C. Then, the secondary antibody goat anti‐rabbit immunoglobulin G (IgG) (ab6785, 1: 1000, Abcam) and horseradish peroxidase (HRP)–labelled streptavidin working solution (0343–10000 U, Yimo Biotechnology Co., Ltd.) were added to the incubation system for a treatment regimen for 20 min at 37°C. The sections were developed using diaminobenzidine (ST033, Guangzhou Whiga Technology Co., Ltd.) and counterstained with haematoxylin (PT001, Bogoo Biological Technology Co., Ltd., Shanghai, China). From each section, five high power fields of vision were randomly selected with 100 cells from each field. Each experiment was conducted three times independently. To evaluate the alveolar fibrosis, mouse lung tissue specimens were sectioned, fixed on glass slides and subjected to HE staining. To further assess the degree of fibrosis, the tissues were sliced into 6‐µm‐thick sections and stained with the Sirius red staining kit (G1470, Beijing Solarbio Science & Technology Co. Ltd.).

### 2.5 Cell culture and treatment

The mouse‐derived alveolar macrophages (AMs) MH‐S cell line (CL‐0597) and 293T cell line (CL‐0005) were provided by (Procell Life Science & Technology Co., Ltd., Wuhan, Hubei, China). Under the conditions of 5% CO2/95% humidified air at 37°C, MH‐S cells were cultured in Roswell Park Memorial Institute 1640 medium supplemented with 0.05 mM β‐mercaptoethanol (Procell Life Science & Technology Co., Ltd.) and 10% fetal bovine serum (Gibco, Invitrogen, USA), while 293T cells were cultured in Dulbecco's modified Eagle medium supplemented with 10% fetal bovine serum (Gibco).

To study the effect of Beclin1 on the autophagy of macrophages, the MH‐S cell line was transfected with the overexpression control (oeCtrl) vector, Beclin1 overexpression (oeBeclin1) vector, small interfering RNA (siRNA) control (siCtrl) or siRNA against Beclin1 (siBeclin1), respectively, and then treated with medium containing crystalline silica (CS; 50 μg/cm^2^), with the addition of normal saline as a negative control (NC) and rapamycin as a positive control, to induce autophagy. The working concentration of the autophagy activator rapamycin (Rapa, HY‐10219, MCE) was 20 nM, while that of the autophagy inhibitor 3‐Methyladenine (3MA, HY‐19312, MCE) was 5 mM and that of the autophagy inhibitor chloroquine (CQ, HY‐17589, MCE) was 20 μM. The MH‐S cells were transfected with oeCtrl, oeE2F1, siCtrl, siE2F1, control‐mimic (Ctrl mimic), miR‐205‐5p mimic, control‐inhibitor (Ctrl inhibitor), miR‐205‐5p inhibitor, oeSKP2, siBeclin1 and their corresponding controls according to the provided instructions of Lipofectamine 2000 (Invitrogen Inc.). The siRNAs were purchased from Ribobio (Guangzhou, China), and the vectors for wild‐type or tagged Beclin1, SKP2 and E2F1 were purchased from GenePharma. Transfection efficiency was detected by a combination of reverse transcription‐quantitative polymerase chain reaction (RT‐qPCR) and Western blot analysis. siRNA was diluted using DEPEC solution to a concentration of 20 µM. Each well was supplemented with 2.5 µl siRNA and 2.5 µl Lipofectamine 2000 with the total volume increased to 200 µl by the addition of Opti‐MEM. Following incubation at room temperature for 5 min, the mixture was added into a 6‐well plate. The plasmid vectors were purchased from GenePharma and used at a dosage of 2 μg.

### 2.6 Cycloheximide (CHX) treatment

MH‐S cells transfected with siCtrl or siSKP2 vector were incubated with 20 μg/ml CHX or solvent control. After 0/30/60/90 min of treatment, the cells were collected for subsequent detection.

### 2.7 RT‐qPCR

The total RNA content was extracted using the TRIzol reagent (15596026, Invitrogen). For mRNA, the mRNA was reversely transcribed into cDNA according to the provided instructions of the PrimeScript RT reagent Kit (RR047A, Takara Bio Inc.). For miRNA, the PolyA tailing detection kit (B532451, Sangon Biotechnology Co. Ltd.) containing the universal PCR primers and universal U6 primers was used to obtain a cDNA library of the miRNA containing PolyA tail. The synthesized cDNA was subjected to treatment with the Fast SYBR Green PCR kit (Applied Biosystems, CA, USA) and ABI PRISM 7300 RT‐PCR system (Applied Biosystems) for qPCR detection, and 3 replicates were set for each well. The relative expression of miRNAs and mRNAs was calculated based on the 2^−ΔΔCt^ method with U6 or β‐actin serving as the internal reference. ΔΔCt = (Ct _target gene_ ‐ Ct _internal reference_) _experimental group_ ‐ (Ct _target gene_ ‐ Ct _internal reference_) _control group_. U6 and miRNA primers were purchased from Ribobio. Other primer designs are shown in Table S1.

### 2.8 Western blot analysis

The cells were collected by trypsinization and lysed with the enhanced radioimmunoprecipitation assay (RIPA) buffer containing specific protease inhibitors (BOSTER Biological Technology Co., Ltd.) followed by protein concentration determination using the bicinchoninic acid protein quantification kit (BOSTER). Proteins were separated by 10% sodium dodecyl sulphate (SDS)‐polyacrylamide gel electrophoresis, after which the separated proteins were electro‐transferred onto polyvinylidene fluoride membranes. The membrane was subjected to a blockade using 5% bovine serum albumin at room temperature for 2 h and probed with the primary antibodies at 4°C overnight and HRP‐conjugated goat anti‐rabbit or goat anti‐mouse secondary antibody for 1 h. After antibody incubation, the membranes were developed using the enhanced chemiluminescence reagents (Millipore, Massachusetts, USA) followed by X‐film exposure. Quantification of the bands was performed using the ImageJ software and normalized to β‐actin. All detailed information regarding the antibodies is shown in Table S2.

### 2.9 Immunoprecipitation (IP)

After trypsinization, the cells (10^7^–10^8^) were collected, lysed by RIPA buffer and centrifuged at 4°C, 7000 g for 20 min followed by collection of the supernatant. Then, 100 µl supernatant was regarded as Input, while the remaining supernatant was incubated with Protein A + G Agarose at 4°C for 1 h for pre‐clearance and centrifuged for transfer of equal volumes of the supernatant to two EP tubes followed by incubation with protein antibody prepared for testing (Table S2) or the corresponding isotype‐matched IgG antibodies, respectively, at 4°C overnight. In the ubiquitination assay, the corresponding plasmids were added in compliance with the above‐mentioned steps with the flag‐labelled anti‐Beclin1/anti‐recombinant Beclin1 antibody in different groups. Then, each tube was incubated with Protein A + G Agarose at 4°C for 1–2 h and centrifuged. The precipitate was rinsed with IP Wash Buffer. The precipitate and Input were added with the corresponding amount of 2 × SDS Buffer, boiled and subjected to Western blot assay.

### 2.10 Dual‐luciferase reporter gene assay

Wild‐type E2F1 3’untranslated region (3’UTR) containing miR‐205‐5p binding sites, mutant‐type E2F1 3’UTR with the mutated miR‐205‐5p binding sites, wild‐type SKP2 promoter containing E2F1 binding sites and mutant‐type SKP2 promoter were synthesized and separately inserted into the pGL3 vector. For miR‐205‐5p/E2F1 binding detection, MH‐S cells were co‐transfected with miR‐205‐5p mimics/E2F1 overexpression vector or controls, mutant‐type pGL3‐E2F1‐3’UTR/pGL3‐SKP2 promoter, wild‐type pGL3‐E2F1‐3’UTR/pGL3‐SKP2 promoter or phRL‐TK, respectively. After 36 h of incubation, the cells were harvested for detection of the luciferase activity with the dual‐luciferase reporter assay system (Promega Corp.). All plasmids were purchased from Addgene.

### 2.11 Chromatin immunoprecipitation (ChIP) assay

Chromatin immunoprecipitation assays were performed using the ChIP kit (Millipore). Briefly, after attaining 70%–80% confluence, the MH‐S cells were fixed with 1% formaldehyde for 10 min and subjected to sonication to isolate the chromatin fragments. Fragmented chromatin was immunoprecipitated overnight with the anti‐RNA polymerase Ⅱ positive control, anti‐human IgG (as NC) or rabbit anti‐E2F1 (Table S2). Protein Agarose/Sepharose was added to precipitate the endogenous DNA‐protein complex. The cross‐linking was reversed overnight at 65°C. RT‐qPCR was performed using these DNA fragments as templates to determine the expression pattern of SKP2. The primer sequences are shown in Table S3.

### 2.12 Detection of autophagic flux

MH‐S cells were treated with the recombinant adenovirus mRFP‐GFP‐LC3 (Hanbio Biotechnology Co., Ltd.)^23^ and then treated with CS or control. Afterwards, the cells were fixed, stained using 4′,6‐diamidino‐2‐phenylindole and observed under a confocal microscope (AMAFD2000, Thermo Fisher Scientific Inc.). The autophagic flux was assessed by counting the numbers of red dots (autolysosomes) and yellow dots (autophagosomes), respectively.

### 2.13 Silicosis mouse model

A total of 92 C57BL/6 mice aged 6–8 weeks purchased from Tangshan People's Hospital Laboratory Animal Center were separately housed in cages at (22–25)°C with 60%–65% humidity under a 12‐h light/dark cycle with free access to food and water. All the mice were fed adaptively for 1 week, and their health status was visually approved prior to the experiment. With 20 mice used as control, silicosis model was established in 72 mice as described previously.^24^ After anaesthesia by an intraperitoneal injection with 50 mg/kg of sodium pentobarbital, 50 μl of CS suspension was injected into mice through the trachea for model establishment. Next, the modelled mice were injected with siBeclin1, miR‐205‐5p agomir (overexpressed miR‐205‐5p), Lenti‐E2F1 (overexpressed E2F1) or Lenti‐SKP2 (overexpressed SKP2) via the tail vein. Furthermore, the mice injected with sterile saline were used as controls.

### 2.14 Statistical analysis

The SPSS 21.0 software (IBM Corporation, Armonk) was adopted for statistical analysis. Measurement data are denoted as mean ± standard deviation. Two groups were compared with the unpaired *t* tests. Multiple groups were compared with one‐way analysis of variance (ANOVA). Comparisons between multiple groups at different time points were performed using two‐way ANOVA. Bonferroni test was adopted for post hoc analysis. Pearson correlation analysis was performed to analyse the correlation between parameters. A value of *p* < 0.05 was considered as statistically significant.

## 3 RESULTS

### 3.1 Inhibiting Beclin1 ameliorates pulmonary fibrosis in mice with silicosis

**FIGURE 1:**
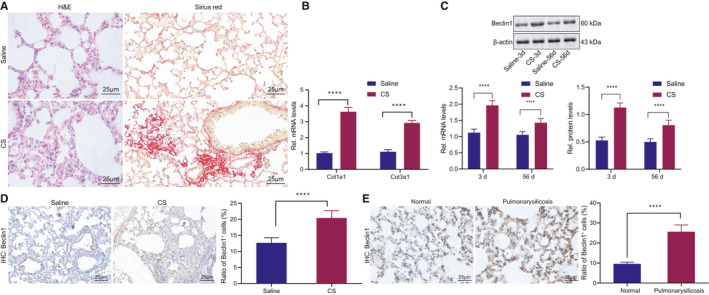
Beclin1 expression was upregulated in the alveolar tissue of mice with CS‐induced silicosis. (A) HE and Sirius red staining of alveolar tissues of mice with CS‐induced silicosis and control mice. (B) RT‐qPCR analysis of Col1a1 and Col3a1 mRNA levels in alveolar tissues of mice with CS‐induced silicosis and control mice. (C) RT‐qPCR and Western blot analysis of Beclin1 mRNA and protein levels in alveolar tissues on day 3 and day 56 after CS induction or saline treatment at the same time points. (D) IHC analysis of Beclin1 in mouse alveolar tissues day 56 after CS treatment. (E) IHC analysis of Beclin1 in clinical diseased tissues of silicosis and normal tissues. Panels A‐E, *p < 0.05, **p < 0.01 and ***p < 0.001, compared with mice treated with saline. Measurement data were analysed by unpaired *t* test between two groups

The involvement of Beclin1 as an autophagy‐related gene in regulating the progression of pulmonary fibrosis has been previously reported,^23^ but its upstream regulatory mechanism remains uncertain. In order to determine the role of Beclin1 in fibrosis after silicosis, we first established a mouse model of silicosis and then determined the severity of fibrosis in alveolar tissues of these model mice by HE and Sirius red staining (Figure 1A). The results showed that CS treatment resulted in inflammatory cell infiltration and alveolar fibrosis. The expression patterns of the collagen genes collagen type I alpha 1 (Col1a1) and Col3a1 in the alveolar tissues were determined by RT‐qPCR, and the Col1a1 and Col3a1 mRNA levels were significantly increased in the alveolar tissues of CS‐treated mice (Figure 1B). The preceding results indicated that the mouse model of silicosis was successfully established.

Next, Beclin1 expression in the alveolus tissues of early‐stage silicosis (day 3 after CS treatment) was observed to be higher than that in the advanced stage silicosis (56 days after CS treatment), and alveolus tissues of silicosis at both stages presented with upregulated Beclin1 expression relative to normal alveolar tissues (Figure 1C). Further, through immunohistochemical staining of the Beclin1 protein in the alveolar tissues on the 56th day after CS treatment, Beclin1‐positive cells were detected to be increased in the silicosis alveolar tissues compared to the normal alveolar tissues (Figure 1D). Consistently, immunohistochemical analysis of clinical samples also indicated that Beclin1‐positive cells of alveolar tissues in stage III silicosis were significantly elevated (Figure 1E). Taken together, these results revealed the upregulated expression of Beclin1 in silicosis, which may be attributed to the lung self‐protective mechanism.

### 3.2 Beclin1 promotes autophagy in AMs

**FIGURE 2:**
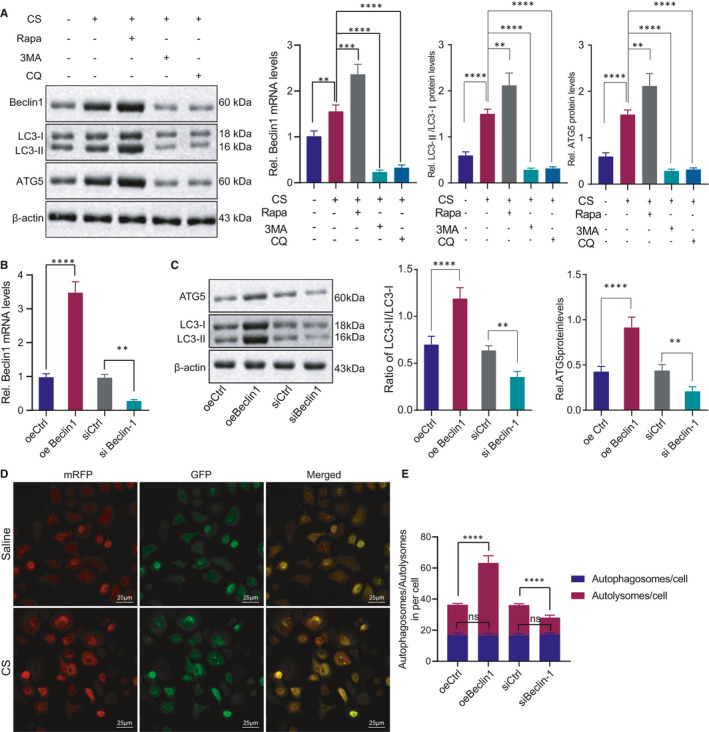
Beclin1 promotes autophagy of CS‐induced AMs. (A) Detection of Beclin1, LC3 and ATG5 in CS‐induced MH‐S cells treated with rapamycin or 3MA. (B) Detection of Beclin1 expression pattern after transfection with oeBeclin1 vector, siBeclin1 or control in MH‐S cells. (C) Detection of LC3 and ATG5 after transfection with oeBeclin1 vector, siBeclin1 or control in MH‐S as well as quantification of LC3‐II/LC3‐I ratio. (D) The distribution of GFP and RFP observed under a confocal microscope after treating MH‐S cells with mRFP‐GFP‐LC3 recombinant adenovirus and CS. (E) The number of autolysosomes/autophagosomes counted under a confocal microscope after the cells in D were with oeBeclin1 vector, siBeclin1 or control followed by CS treatment. *p < 0.05; **p < 0.01; ***p < 0.001 and ****p < 0.0001. Measurement data were analysed by unpaired *t* test between two groups. The experiment was conducted 3 times independently

Beclin1 engages in regulating cellular autophagy as a functional component of the phosphatidylinositol‐3‐kinase complex, and numerous studies have identified the protective effect of autophagy in fibrotic diseases.^25^, ^26^ In order to determine whether the Beclin1 expression pattern in AMs changed abnormally, we treated the MH‐S cell line with CS in combination with the autophagy activator rapamycin or autophagy inhibitor 3MA. Our findings revealed that the mRNA and protein levels of Beclin1, LC3 and autophagy‐related gene‐5 (ATG5) were increased after treatment with CS, and Beclin1, LC3 and ATG5 increased upon combination treatment with CS and rapamycin. However, Beclin1 decreased by 3MA in CS‐induced MH‐S cells (Figure 2A). The above results indicated that Beclin1 may induce autophagy in AMs. In order to determine the promoting effect of Beclin1 on the autophagy of AMs, we initially transfected oeBeclin1 vector, siBeclin1 or controls into MH‐S cells followed by analysis of the transfection efficiency at the mRNA and protein levels (Figure 2B). Then, the autophagy‐related proteins LC3 and ATG5 in lung tissues were measured by Western blot analysis, the results of which revealed that the overexpression of exogenous Beclin1 promoted LC3 and ATG5 expression, and reduced LC3 and ATG5 expression patterns were induced by Beclin1 silencing (Figure 2C). Next, the MH‐S cell lines were injected with mRFP‐GFP‐LC3 recombinant adenovirus followed by CS treatment. After CS treatment, the counts of LC3 puncta and mRFP fluorescent spots increased significantly (Figure 2D). Furthermore, our findings highlighted that the overexpression of Beclin1 increased the numbers of LC3 puncta and mRFP fluorescent spots, with a conflicting consequent of Beclin1 silencing (Figure 2E). The afore‐mentioned results demonstrated that Beclin1 stimulated the autophagy in AMs.

### 3.3 K48‐linked poly‐ubiquitination of Beclin1 by SKP2 promotes Beclin1 degradation

**FIGURE 3:**
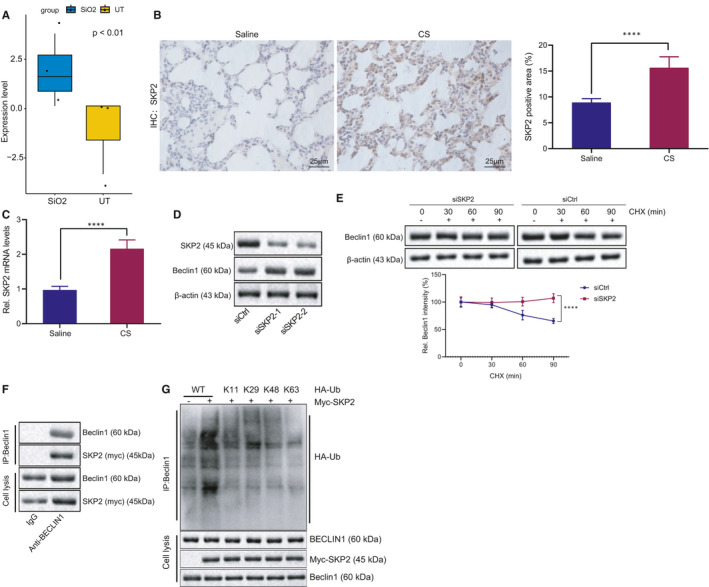
SKP2 enhances the K48‐linked poly‐ubiquitination of Beclin1. (A) The expression pattern of SKP2 in microarray data set GSE110711 of mice with silicosis in GEO database, with SiO_2_ representing silicosis model and UT representing the control. (B) IHC analysis of SKP2 in alveolar tissues of mice treated with CS or saline. C, RT‐qPCR analysis of SKP2 in alveolar tissues of mice treated with CS or saline. (D) Detection of SKP2 and Beclin1 protein after MH‐S cells were transfected with siCtrl or siSKP2. (E) Detection of SKP2 and Beclin1 protein after MH‐S cells were transfected with siCtrl or siSKP2 and treated with protein synthesis inhibitor CHX. (F) Detection of Beclin1 and myc‐SKP2 proteins after overexpressing myc‐SKP2 in the MH‐S cell line for Co‐IP using Beclin1 antibody. (G) Detection of Beclin1, myc‐SKP2 and HA‐Ub using Beclin1 antibody after MH‐S cells were transfected with vectors encoding myc‐SKP2 and either wild‐type HA‐tagged ubiquitin or a lysine‐less mutant of HA‐tagged ubiquitin (Ub). *p < 0.05 vs. MH‐S cells treated with siCtrl (panel E); **p < 0.01 vs. normal mice (UT) (panel A) or mice treated with saline (panel B); and ***p < 0.001 vs. mice treated with saline (panel C). Measurement data were analysed by unpaired *t* test between two groups. Comparisons between multiple groups with different time points were performed using two‐way ANOVA. Bonferroni test was used for post hoc analysis. The experiment was conducted 3 times independently

As SKP2 might promote the ubiquitination of Beclin1,^21^ we initially analysed the microarray data set GSE110711 of mice with silicosis in the GEO database by R language and identified that the SKP2 gene was highly expressed in the setting of pulmonary fibrosis (Figure 3A). Immunohistochemical staining suggested that the SKP2‐positive cells were significantly increased in the alveolar tissues of mice with early silicosis compared to the normal alveolar tissues (Figure 3B). To further verify whether SKP2 was elevated in early silicosis, we analysed the SKP2 mRNA levels in the afore‐mentioned tissues by RT‐qPCR and confirmed the presence of a significantly increased SKP2 expression pattern in the alveolar tissues of early silicosis (Figure 3C). We then identified a decreased SKP2 expression pattern in the MH‐S cells transfected with siSKP2 (Figure 3D). After treatment with the protein synthesis inhibitor CHX, the stability of Beclin1 protein was increased in response to SKP2 silencing (Figure 3E). Additionally, IP confirmed that SKP2 and Beclin1 could explicitly interact with each other (Figure 3F). Then, MH‐S cells were transfected with vectors encoding SKP2 and either wild‐type HA‐tagged ubiquitin or a lysine‐less mutant of HA‐tagged ubiquitin. The results revealed that SKP2 could radically induce K48‐linked poly‐ubiquitination of Beclin1 (Figure 3G). Altogether, SKP2 could enhance the poly‐ubiquitination of Beclin1 and thus augment the ubiquitin‐mediated degradation of Beclin1.

### 3.4 E2F1 binds to the SKP2 promoter and upregulates its expression

**FIGURE 4:**
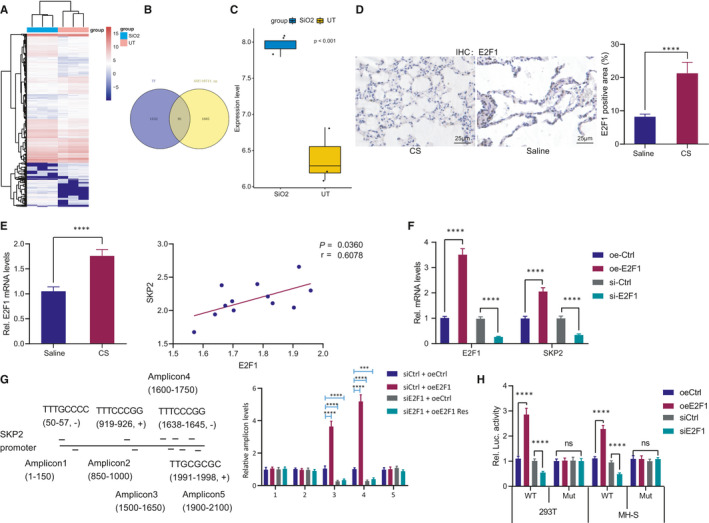
E2F1 is a transcription factor of SKP2. (A) Heat map of differentially expressed genes in microarray data set GSE110711 of mice with silicosis, with SiO_2_ representing silicosis model and UT representing the control. (B) The intersection of the gene upregulated in microarray data set GSE110711 and 1623 mouse transcription factors obtained from AnimalTFDB. (C) Expression pattern of E2F1 in microarray data set GSE110711. (D) IHC analysis of E2F1 in alveolar tissues of mouse models. (E) RT‐qPCR analysis of E2F1 in alveolar tissues of mouse models, and correlation between E2F1 and SKP2 expression pattern. (F) Detection of E2F1 and SKP2 mRNA level after MH‐S cells transfected with E2F1 overexpression vector or siRNA. (G) Five pairs of amplicons were designed for binding site of E2F1 on the SKP2 promoter based on MotifMap, followed by detection of amplicon binding after transfection of siE2F1 or oeE2F1 vector with mutant binding site in MH‐S cells. (H) Detection of the luciferase activity after co‐transfection of luciferase reporter gene vector and siE2F1 or oeE2F1 in 293T and MH‐S cells. *p < 0.05 vs. alveolar tissues in mice treated with saline (panel D); **p < 0.01 vs. MH‐S cells treated with siCtrl (panels F) or 293T/MH‐S cells treated with siCtrl (panel H); and ***p < 0.001 vs. normal mice (UT) (panel C), mice treated with saline (panel E), MH‐S cells treated with oeCtrl/siCtrl (panel F), MH‐S cells treated with siCtrl +oeCtrl (panel G) or 293T/MH‐S cells treated with oeCtrl (panel H); n.s., no significance. Measurement data were analysed by unpaired *t* test between two groups. Correlation between two parameters was determined by Pearson correlation. The experiment was conducted 3 times independently

To further explore the upstream mechanism of SKP2, we initially analysed the microarray data set GSE110711 in the GEO database using R language and screened out 3246 differentially expressed genes in pulmonary fibrosis (Figure 4A). Additionally, 1623 mouse transcription factors were provided by AnimalTFDB. By intersecting them and the upregulated genes obtained by microarray data set analysis, 91 key transcription factors were identified (Figure 4B).

Next, we screened E2F1 as a potential transcription factor for SKP2 through the hTFtarget database. R analysis showed that E2F1 gene was highly expressed in pulmonary fibrosis in the microarray data set GSE110711 (Figure 4C). As shown by IHC, E2F1‐positive cells were increased notably in the alveolar tissues of mice with silicosis (Figure 4D). RT‐qPCR analysis revealed a significantly increased E2F1 expression pattern in silicosis, which was highly positively correlated with the expression pattern of SKP2 (Figure 4E). RT‐qPCR showed that the E2F1 overexpression significantly increased the mRNA level of SKP2, whereas E2F1 silencing reduced the mRNA level of SKP2 (Figure 4F). Next, we designed 5 pairs of amplicons for E2F1 potential binding site in the SKP2 promoter based on MotifMap. ChIP‐PCR analysis of the MH‐S cell line identified that the SKP2 promoter sequence TTCCCGG, spanning from 1638 to 1645, was the potential binding site of E2F1. Moreover, siE2F1 could significantly reduce the enrichment of E2F1 in the SKP2 promoter region (Figure 4G). To validate this finding, dual‐luciferase reporter gene assay was conducted in the 293T and MH‐S cells, which revealed that overexpression of E2F1 significantly promoted the luciferase activity of the wild‐type SKP2 promoter sequence, whereas E2F1 inhibition was indicative of a conflicting effect. Furthermore, either promoting or inhibiting E2F1 expression had no significant effect on the luciferase activity of the mutant SKP2 promoter sequence (Figure 4H). Based on these results, it was rationale to conclude that E2F1 could potentially bind to the SKP2 promoter and upregulate the SKP2 expression.

### 3.5 miR‐205‐5p targets E2F1 and inhibits its expression

**FIGURE 5:**
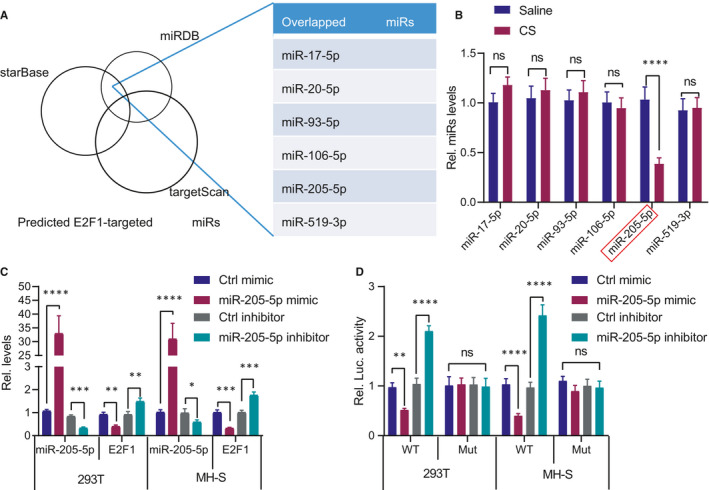
E2F1 is the potential target of miR‐205‐5p. (A) Screening of miRNAs potentially regulating E2F1 through starBase, TargetScan and miRDB database. (B) Quantification of miR‐17‐5p/miR‐20‐5p/miR‐93‐5p/miR‐106‐5p/miR‐205‐5p/miR‐519‐3p in alveolus tissues of mice with silicosis. **p < 0.01 vs. mice treated with saline; n.s., no significance. (C) Quantification of miR‐205‐5p and E2F1 after transfection of miR‐205‐5p mimic or inhibitor in 293T and MH‐S cells. **p < 0.01 and ***p < 0.001 vs. 293T/MH‐S cells treated with Ctrl mimic/inhibitor. (D) Detection of the luciferase activity after co‐transfection of luciferase reporter gene vector and miR‐205‐5p mimic or inhibitor in 293T and MH‐S cells. **p < 0.01 and ***p < 0.001 vs. 293T/MH‐S cells treated with Ctrl mimics/inhibitor; n.s., no significance. Measurement data were analysed by unpaired *t* test between two groups. The experiment was conducted 3 times independently

Furthermore, we screened the miRNAs potentially regulating E2F1 from the starBase, TargetScan and miRDB database and identified E2F1 as a potential target gene of miR‐17‐5p/miR‐20‐5p/miR‐93‐5p/miR‐106‐5p/miR‐205‐5p/miR‐519‐3p (Figure 5A). The quantitative analysis of miRNAs in the mouse silicosis tissues identified that only the miR‐205‐5p expression pattern was significantly downregulated in the alveolar tissues of silicosis (Figure 5B). To verify the regulatory effect of miR‐205‐5p on E2F1, miR‐205‐5p mimic or inhibitor was transfected into the 293T and MH‐S cell lines. The results indicated that miR‐205‐5p mimic could significantly reduce the mRNA level of E2F1, whereas the miR‐205‐5p inhibitor resulted in an elevated E2F1 mRNA level (Figure 5C). To demonstrate that miR‐205‐5p directly binds to E2F1 UTR, dual‐luciferase reporter gene assay was performed. The results revealed that miR‐205‐5p mimic could significantly inhibit the luciferase activity of the wild‐type E2F1 3'UTR, whereas the miR‐205‐5p inhibitor led to an opposite effect (Figure 5D). Altogether, miR‐205‐5p interacted with E2F1 to negatively regulate its expression.

### 3.6 miR‐205‐5p promotes AM autophagy *via* E2F1/SKP2/Beclin1 axis

**FIGURE 6:**
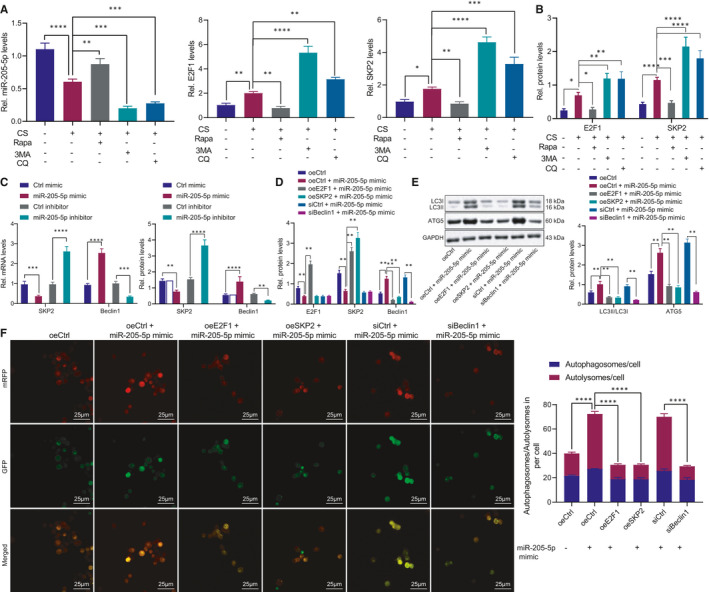
The E2F1/SKP2/Beclin1 axis is a necessary link for miR‐205‐5p to promote autophagy of AMs. A‐B, The expression patterns of miR‐205‐5p, E2F1 and SKP2 mRNAs (A) as well as E2F1 and SKP2 proteins (B) in CS‐induced MH‐S cells treated with rapamycin or 3MA. C, Detection of SKP2 and Beclin1 in MH‐S cells after transfection with miR‐205‐5p mimic or inhibitor. D‐E, Detection of E2F1, SKP2 and Beclin1 (D) as well as LC3‐II/LC3‐I ratio and ATG5 (E) in MH‐S cells transfected with oeE2F1/oeSKP2 or siBeclin1 and miR‐205‐5p mimic. (F) Quantification of the numbers of LC3 puncta and mRFP fluorescent spots after CS‐induced MH‐S cells were treated with recombinant adenovirus mRFP‐GFP‐LC3, miR‐205‐5p mimic, oeE2F1/oeSKP2 vectors, or siBeclin1. *p < 0.05; **p < 0.01; ***p < 0.001; and ****p < 0.0001. Measurement data were analysed by unpaired *t* test between two groups and one‐way ANOVA among multiple groups. The experiment was repeated 3 times independently

To identify the regulatory role of the miR‐205‐5p/E2F1/SKP2/Beclin1 axis in autophagy of AMs, the MH‐S cells were treated with CS and combined with rapamycin or 3MA. The obtained results revealed that the enhanced autophagy was supported by increased miR‐205‐5p level as well as reduced E2F1 and SKP2 levels (Figure 6A,B). In addition, miR‐205‐5p mimic treatment led to decreased SKP2 and increased Beclin1 in MH‐S cells, while miR‐205‐5p inhibitor led to elevated SKP2 and reduced Beclin1 at the mRNA and protein levels (Figure 6C). Next, we co‐transfected oeE2F1/oeSKP2 vector or siBeclin1 in MH‐S cells with miR‐205‐5p mimic, respectively. The findings revealed that miR‐205‐5p mimic reduced the expression patterns of E2F1 and SKP2 and increased the expression pattern of Beclin1, while overexpression of E2F1/SKP2 could reverse the increase in Beclin1 expression pattern induced by miR‐205‐5p (Figure 6D). Moreover, increased LC3 and ATG5 were evident in MH‐S cells with miR‐205‐5p mimic, while overexpressing E2F1 or SKP2 or inhibiting Beclin1 reversed the effect of miR‐205‐5p mimic (Figure 6E). Finally, after treatment of the above cells with the recombinant adenovirus mRFP‐GFP‐LC3 and CS, we identified that the miR‐205‐5p mimic significantly elevated the numbers of LC3 puncta and mRFP fluorescent spots, which could be reversed by overexpressing E2F1 or SKP2 or inhibiting Beclin1 (Figure 6F). Concisely, E2F1/SKP2/Beclin1 axis was the mediator for miR‐205‐5p to promote AM autophagy.

### 3.7 miR‐205‐5p inhibits pulmonary fibrosis *via* E2F1/SKP2/Beclin1 axis

**FIGURE 7:**
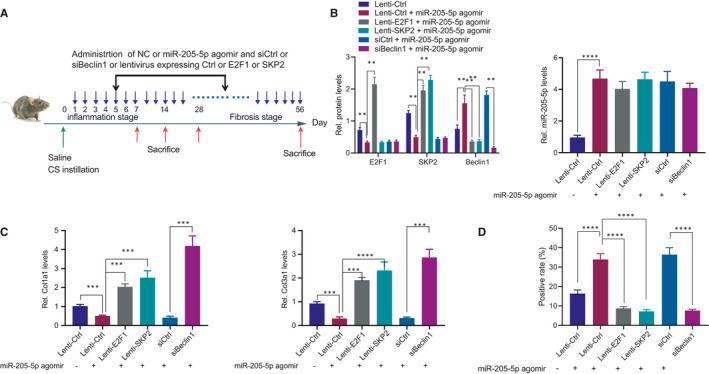
The E2F1/SKP2/Beclin1 axis was a necessary link for miR‐205‐5p to suppress pulmonary fibrosis. (A) Model diagram of silicosis induction in mice and injection with agomir, Lenti vectors or siRNAs. (B) Detection of miR‐205‐5p expression pattern, and E2F1, SKP2 and Beclin1 protein expression patterns in mouse models. (C) Detection of Col1a1/Col3a1 mRNA expression pattern in mouse models. (D) Detection of LC3 protein expression pattern in the mouse model. *p < 0.05; **p < 0.01; ***p < 0.001; and ****p < 0.0001. Measurement data were analysed by unpaired *t* test between two groups and one‐way ANOVA among multiple groups

Finally, the silicosis mouse models were established for further investigation of the regulatory mechanisms concerning miR‐205‐5p in silicosis. After successful model establishment, the mice were injected with NC agomir +Lenti‐Ctrl, miR‐205‐5p agomir +Lenti‐Ctrl, miR‐205‐5p agomir +Lenti‐E2F1, miR‐205‐5p agomir +Lenti‐SKP2, miR‐205‐5p agomir +siCtrl and miR‐205‐5p agomir +siBeclin1, respectively (Figure 7A). The efficiency of Lenti‐E2F1, Lenti‐SKP2 and siBeclin1 was validated by Western blot analysis (Figure 7B). The findings revealed that miR‐205‐5p agomir significantly reduced the protein levels of E2F1 and SKP2 along with an elevated Beclin1 protein level, all of which were reversed by subsequent delivery of Lenti‐E2F1. However, treatment with both miR‐205‐5p agomir and Lenti‐SKP2 did not significantly alter the E2F1 protein level, increased the SKP2 protein level and decreased the Beclin1 protein level when compared with the miR‐205‐5p agomir treatment alone. In addition, no significant difference was evident regarding the protein levels of E2F1 and SKP2, yet the Beclin1 protein level was reduced after treatment with both miR‐205‐5p agomir and siBeclin1 in comparison with miR‐205‐5p agomir treatment alone. Additionally, miR‐205‐5p agomir suppressed the pulmonary fibrosis in mice with silicosis, while Lenti‐E2F1, Lenti‐SKP2 or siBeclin1 counteracted this suppression (Figure S1A). Moreover, the mice with silicosis following treatment of miR‐205‐5p agomir showed upregulated expression patterns of Col1a1, Col3a1 and LC3, while this upregulation was annulled by Lenti‐E2F1, Lenti‐SKP2 or siBeclin1 (Figure 7C,D, Supplementary Figure 1B,C). Therefore, the afore‐mentioned findings revealed that miR‐205‐5p inhibited pulmonary fibrosis *via* the E2F1/SKP2/Beclin1 axis.

## 4 DISCUSSION

**FIGURE 8:**
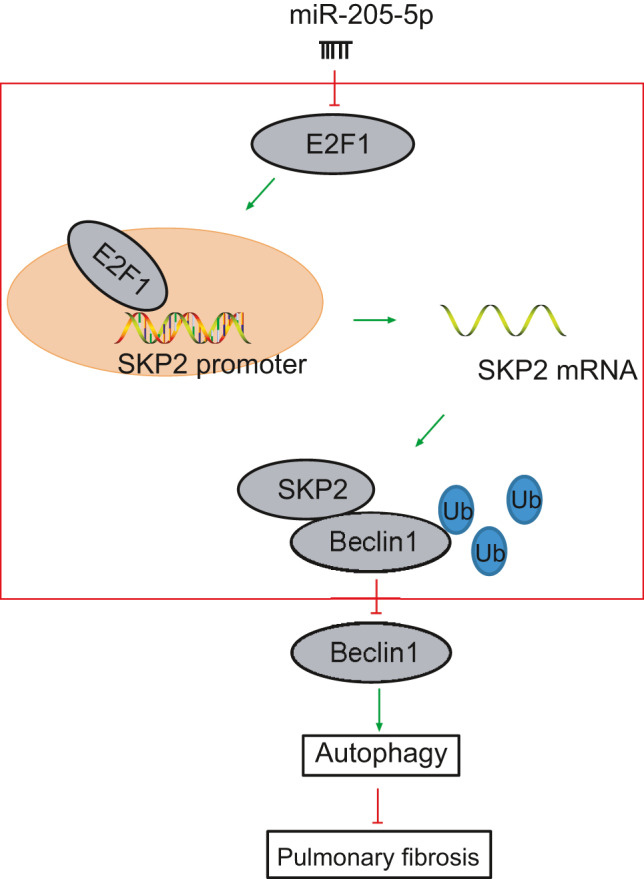
The mechanism concerning miR‐205‐5p‐targeted E2F1 in CS‐induced silicosis. miR‐205‐5p impaired SKP2‐mediated poly‐ubiquitination of Beclin1 by targeting E2F1. Additionally, our findings demonstrated the protective effect of miR‐205‐5p against silicosis and suggested that miR‐205‐5p/E2F1/SKP2/Beclin1 axis might be a therapeutic target

In the current study, we analysed the regulatory molecule upstream of Beclin1 by investigation of numerous databases and identified the miR‐205‐5p/E2F1/SKP2 circuitry as the prime target. The findings showed that miR‐205‐5p positively regulated the autophagy of AMs. Mechanistically, miR‐205‐5p suppressed the SKP2‐mediated poly‐ubiquitination of Beclin1 by interacting with E2F1 (Figure 8). These observations demonstrated the protective effect of miR‐205‐5p against silicosis and suggested the involvement of miR‐205‐5p/E2F1/SKP2/Beclin1 axis in autophagy and pulmonary fibrosis.

Recently, autophagy was identified as a novel defence mechanism for lung injury induced by different factors, including CS.^27^, ^28^ A prior study reported the ability of enhancement of autophagy of AMs to reduce CS‐induced lung inflammation and fibrosis.^23^ Beclin1, the initially discovered mammalian autophagy‐related gene, could evidently inhibit pulmonary fibrosis in silicosis.^20^ In this study, our findings unravelled enhanced autophagy of AMs in response to silencing Beclin1.

Accumulating evidence has elicited the ability of miRNA to bind to transcription factors and regulate gene expression, coherently mediating cell function, thus establishing significance in various diseases.^29^ The current study demonstrated that miR‐205‐5p was poorly expressed in mice with silicosis, which was negatively correlated with the E2F1 expression pattern. Additionally, miR‐205‐5p suppressed pulmonary fibrosis by inducing the autophagy of AMs. Similarly, it has been previously documented that miR‐205 is poorly expressed in pulmonary fibrosis and its upregulation can delay pulmonary fibrosis.^30^ E2F1, an established transcription factor in cell cycle regulation, exerts tumour‐suppressive activity and anti‐proliferative properties.^31^ By targeting E2F1, miR‐205 inhibits melanoma cell proliferation and induces senescence^32^ or enhances the cisplatin sensitivity of glioma cells.^33^ miR‐205‐5p and miR‐342‐3p synergistically inhibit the transcription factor E2F1 to reduce anti‐cancer chemotherapy resistance.^34^ On the basis of these findings, we identified the direct binding relationship of miR‐205‐5p to E2F1 3’UTR.

E2F1 is a member of the E2F transcription factor family, and this factor has been extensively studied due to its involvement in cell cycle regulation.^35^, ^36^, ^37^ E2F1, often dysregulated and activated in human cancers, is inversely associated with the survival of patients.^32^, ^38^, ^39^ Accumulating studies have reported that the downregulation of E2F1 leads to high levels of autophagy with regulatory involvement of the transcription of autophagy genes.^40^, ^41^ For instance, amino acid deprivation‐induced autophagy upregulates DIRAS3 by reducing E2F1 and E2F4 transcriptional repression.^42^ Resveratrol‐mediated disruption of E2F1 induces autophagy and inhibits apoptosis to inhibit doxorubicin‐induced cardiotoxicity.^43^ Recent studies have characterized SKP2 as the transcription target of E2F1.^44^, ^45^ Additionally, E2F1 and SKP2 are highly expressed in multiple tumour cells collaboratively.^46^, ^47^, ^48^ E2F1 transcription factor is engaged in the SKP2 promoter to stimulate the proliferation of breast cancer cells^49^ or non‐small cell lung cancer.^50^ In this study, our findings revealed that E2F1 was enriched in the SKP2 promoter region and promoted the expression of SKP2.

SKP2, a vital cell cycle regulator, engages cyclin and other substrate proteins to facilitate degradation by ubiquitination.^51^ Recently, SKP2 was reported as a potential molecular target for human pulmonary fibrosis as its inhibition reduces the pulmonary fibrosis in silicosis.^52^ Additionally, SKP2 negatively regulates autophagy in hypertrophic cardiomyocytes.^53^ Notably, K48‐linked poly‐ubiquitination of Beclin1 by SKP2 suppresses the autophagic flow.^21^ The regulation of Beclin1 through post‐translational modification has been well documented, with involvement of phosphorylation and ubiquitination.^54^ In addition to the K48‐linked poly‐ubiquitination of Beclin1 at K402 by SKP2, Beclin1 also undergoes ubiquitination by additional E3 ligases, in different locations and different types of poly‐ubiquitination.^55^, ^56^, ^57^ According to reports, modified Beclin1 protein in the fibroblasts during idiopathic pulmonary fibrosis is associated with autophagy dysfunction.^58^ Here, we identified that SKP2 could evidently facilitate K48‐linked poly‐ubiquitination of Beclin1. Finally, the cell and murine models suggested that miR‐205‐5p impeded SKP2‐mediated Beclin1 ubiquitination to promote Beclin1‐mediated autophagy and suppress pulmonary fibrosis.

In conclusion, our findings revealed the pro‐autophagic and anti‐fibrotic effects of miR‐205‐5p in silicosis. Our research hence suggests that miR‐205‐5p can be regarded as a promising target for the treatment of silicosis in the future. However, more investigations are warranted before clinical application.

## CONFLICTS OF INTEREST

All authors declare that they have no conflicts of interest.

## AUTHOR CONTRIBUTION

**Qingzeng Qian:** Conceptualization (equal). **Qinghua Ma:** Data curation (equal). **Bin Wang:** Formal analysis (equal). **Qingqiang Qian:** Funding acquisition (equal). **Changsong Zhao:** Formal analysis (equal). **Fumin Feng:** Validation (equal). **Xiaona Dong:** Conceptualization (equal).

## Supporting information

Figure S1

Table S1‐S3

## Data Availability

All data included in this study are available upon request by contact with the corresponding author.
