# Supplementary figures and images for: MicroRNA‐205‐5p targets E2F1 to promote autophagy and inhibit pulmonary fibrosis in silicosis through impairing SKP2‐mediated Beclin1 ubiquitination
Source: J Cell Mol Med. 2021 Aug 24;25(19):9214–27. doi: 10.1111/jcmm.16825 (PMC8500965; doi:10.1111/jcmm.16825)

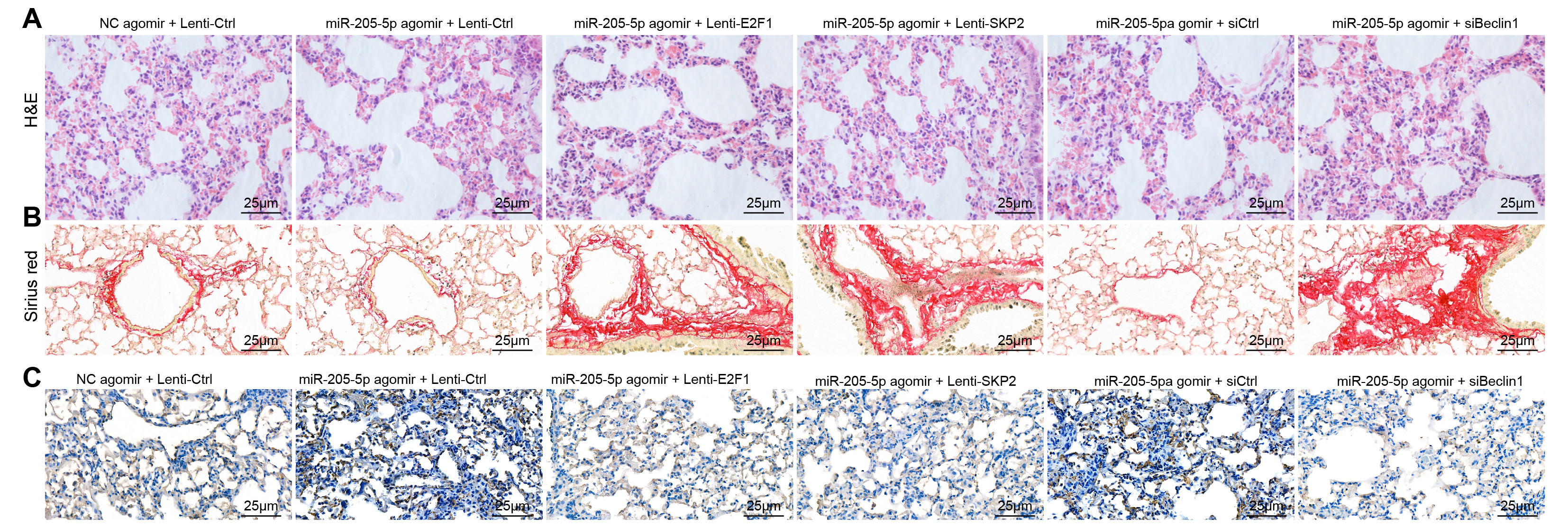

Supplement: Supplementary file 1 — Figure S1 [file JCMM-25-9214-s002.jpg]
