# Supplementary material for: MicroRNA‐205‐5p targets E2F1 to promote autophagy and inhibit pulmonary fibrosis in silicosis through impairing SKP2‐mediated Beclin1 ubiquitination
Source: J Cell Mol Med. 2021 Aug 24;25(19):9214–27. doi: 10.1111/jcmm.16825 (PMC8500965; doi:10.1111/jcmm.16825)
Supplement: Supplementary file 2 — Table S1‐S3 [file JCMM-25-9214-s001.docx]

**Supplementary Table 1** Primer sequences for RT-qPCR

| Gene name | Primer sequence |
| --- | --- |
| miR-17-5p | F: 5’-CACATCATCAAAGTGCTTACAGTG-3’ |
|  | R: 5’-GTGCAGGGTCCGAGGT-3’ |
| miR-20-5p | F: 5’-TAAAGTGCTTATAGTGCAGGTAG-3’ |
|  | R: 5’-GTGCAGGGTCCGAGGT-3’ |
| miR-93-5p | F: 5’-GCCGCCAAAGTGCTGTTC-3’ |
|  | R: 5’-CAGAGCAGGGTCCGAGGTA-3’ |
| miR-106-5p | F: 5’-ATGGAGGGGTCTAAGGCGTC-3’ |
|  | R: 5’-TGGGCTGTGGTAAGTAATGGA-3’ |
| miR-205-5p | F: 5’-TCCTTCATTCCACCGGAGTCTG-3’ |
|  | R: 5’-GCGAGCACAGAATTAATACGAC-3’ |
| miR-519-3p | F: 5’-GCGCAAAGTGCATCCTTTTA-3’ |
|  | R: 5’-GTGCAGGGTCCGAGGT-3’ |
| U6 | F: 5’-GCTTCGGCAGCACATATACTAAAAT-3’ |
|  | R: 5’-CGCTTCACGAATTTGCGTGTCAT-3’ |
| Beclin1 | F: 5’-ATGGAGGGGTCTAAGGCGTC-3’ |
|  | R: 5’-TGGGCTGTGGTAAGTAATGGA-3’ |
| E2F1 | F: 5’-GAGAAGTCACGCTATGAAACCTC-3’  F: 5’-CCCAGTTCAGGTCAACGACAC-3’ |
| SKP2 | F: 5’-CCTCCAAGGAAACGAGTCAAG-3’ |
|  | R: 5’-CAGGAGACACCTGGAAAGTTC-3’ |
| Col1a1 | F: 5’-GAGGGCCAAGACGAAGACATC-3’ |
|  | R: 5’-CAGATCACGTCATCGCACAAC-3’ |
| Col3a1 | F: 5’-GGAGCTGGCTACTTCTCGC-3’ |
|  | R: 5’-GGGAACATCCTCCTTCAACAG-3’ |
| β-actin | F: 5’-ATCATGAAGTGTGACGTGGACAT-3’ |
|  | R: 5’-AGGAGCAATGATCTTGATCTTCA-3’ |

Note: RT-qPCR, reverse transcription quantitative polymerase chain reaction; miR, microRNA; E2F1, E2F transcription factor 1; SKP2, S-phase kinase-associated protein 2; Col1a1, collagen type I alpha 1; F, forward; R, reverse.

**Supplementary Table 2** Antibodies

| Antibody | Specie | Number | Manufacturer | Producing area | Antibodies uses | Dilution ratio | | |
| --- | --- | --- | --- | --- | --- | --- | --- | --- |
|  |  |  |  |  |  | WB | IHC | ChIP |
| β-actin | rabbit | ab179467 | Abcam | UK | Western blot | 1:5000 | - | - |
| ATG5 | rabbit | 10181-2-AP | Proteintech | China | Western blot | 1:1000 | - | - |
| LC3 | rabbit | ab48394 | Abcam | UK | Western blot/IHC | 1:1000 | 1:400 | - |
| BECLIN1 | rabbit | ab210498 | Abcam | UK | IHC/Western blot | 1:1000 | 1:100 | - |
| E2F1 | rabbit | ab179445 | Abcam | UK | ChIP/IHC/WB | 1:1000 | 1:250 | 1:50 |
| SKP2 | rabbit | ab68455 | Abcam | UK | IHC/WB | 1:1000 | 1:500 | - |
| MYC-tag | mouse | 60003-2-Ig | Proteintech | China | WB | 1:10000 | - | - |
| FLAG-tag | mouse | 66008-3-Ig | Proteintech | China | WB | 1:1000 | - | - |
| HA-tag | mouse | 66006-2-Ig | Proteintech | China | WB | 1:10000 | - | - |
| Secondary antibody | mouse | ab205719 | Abcam | UK | WB | 1:10000 | - | - |
| Secondary antibody | rabbit | ab6721 | Abcam | UK | WB | 1:10000 | - | - |
| IgG | rabbit | ab6785 | Abcam | UK | IHC | - | 1:1000 | - |

Note: WB, Western blot; IHC, immunohistochemistry; LC3, light chain 3; ATG5, autophagy related 5; E2F1, E2F transcription factor 1; SKP2, S-phase kinase-associated protein 2; IgG, immunoglobulin G; ChIP, chromatin immunoprecipitation.

**Supplementary Table 3** Primer sequences for ChIP

| Primer name | Primer sequence |
| --- | --- |
| Amplicon1 | F: 5’-ATTCCCATATTAGGAAGAT-3’ |
|  | R: 5’-CTGGGAACTAGAATACTTGCA-3’ |
| Amplicon2 | F: 5’-TTATGAGGCTTAGAATTCAT-3’ |
|  | R: 5’-AAGAGTAGGGAGCGTGACA-3’ |
| Amplicon3 | F: 5’-GCCACTCGGGGTCTGCCGGGT-3’ |
|  | R: 5’-ACCTGGACAGCTGTGGCG-3’ |
| Amplicon4 | F: 5’-GATCTCGCCAGACAGCGTCTG-3’ |
|  | R: 5’-GCCGCTGGACTGTAGACGGA-3’ |
| Amplicon5 | F: 5’-GAAGGAAGTAAGGGCGAGC-3’ |
|  | R: 5’-GCTGCTCGCCTCCCAGATTCC-3’ |

Note: ChIP, chromatin immunoprecipitation; F, forward; R, reverse.
